# Global Research Trends in the Links between Periodontal Disease and Cancer: A Bibliometric Analysis

**DOI:** 10.3390/pathogens13090789

**Published:** 2024-09-12

**Authors:** Suh-Woan Hu, Jaw-Ji Yang, Yuh-Yih Lin

**Affiliations:** 1 Institute of Oral Sciences, College of Oral Medicine, Chung Shan Medical University, Taichung 40201, Taiwan; suhwoan@csmu.edu.tw (S.-W.H.); jjyang@csmu.edu.tw (J.-J.Y.); 2 Department of Stomatology, Chung Shan Medical University Hospital, Taichung 40201, Taiwan; 3 School of Dentistry, College of Oral Medicine, Chung Shan Medical University, Taichung 40201, Taiwan

**Keywords:** periodontal diseases, neoplasms, bibliometrics, databases, bibliographic

## Abstract

Both periodontal disease and cancer are prevalent conditions with significant impacts on individuals and society. Extensive research has suggested a potential link between these two diseases. This study conducted a bibliometric analysis using the Thomson Reuters Web of Science Core Collection database, focusing on publications from 2014 to 2023. The analysis included data extraction and examination of authors, affiliations, publication dates, journals, countries, citation counts, keywords, and the H-index. A total of 253 relevant articles were identified, showing an increasing trend in both publications and citations over the years. The analysis highlighted the most productive authors, institutions, and countries/regions, with Michaud DS and Abnet CC leading in the number of publications. Highly cited articles emphasized the role of specific oral microbiota, particularly *F. nucleatum* and *P. gingivalis*, in various cancers, suggesting their potential as diagnostic markers and therapeutic targets. Four key thematic clusters emerged from the keyword analysis: the broader health implications of periodontal disease, the microbiome’s role in carcinogenesis, inflammation, and specific bacteria in cancer, and epidemiological methods in studying the disease–cancer association. This bibliometric analysis underscores the growing interest in the connection between periodontal disease and cancer. Future research should adopt interdisciplinary approaches, focus on large-scale microbiome studies and longitudinal research to understand the systemic effects of periodontal disease, identify cancer-associated bacterial profiles, and investigate the molecular mechanisms of bacterial carcinogenesis. Additionally, public health interventions aimed at improving oral hygiene and reducing cancer risk factors are recommended.

## 1. Introduction

Periodontal disease is one of the most prevalent oral diseases, and it significantly impacts individuals and society [1]. According to the Global Oral Health Status Report 2022, severe periodontal disease affected approximately 1.08 billion people worldwide in 2019, with an age-standardized prevalence of 18.82% among individuals over 15 years old [2]. Cancer remains the foremost cause of premature death worldwide, ranking first in 57 countries and second in 70 [3]. The latest analysis of GLOBOCAN 2020 estimates indicated approximately 19.29 million new cancer cases in 2020. Additionally, it revealed 9.96 million cancer-related deaths within the same year [4]. Many epidemiological studies and reviews have reported a significant association between periodontal disease and cancer incidence and/or mortality among different populations after adjusting for confounding factors [5,6,7,8,9,10,11,12,13,14,15,16,17,18]. Several studies have investigated the biological mechanisms linking periodontal disease and pathogens to tumorigenesis. They suggested that the mechanism might involve several factors, including chronic inflammation [18,19,20], interference with cellular signaling pathways promoting cell survival and proliferation [12,21], production of bacterial metabolic by-products that caused DNA damage and mutagenesis [12], and systemic immune modulation [22,23].

Given the substantial impact of periodontal disease and cancer on individuals and society, as well as the abundant literature on the periodontal disease–cancer relationship, it is crucial to identify the global research trends and hotspots. Bibliometric analysis can help researchers depict the evolving knowledge structure and trends within a research field over time [24]. Bibliometric research has gained increasing popularity across various academic disciplines. Despite numerous studies investigating the link between periodontal disease and cancer, a comprehensive bibliometric analysis on this specific topic has not been fully explored. This study aims to conduct a bibliometric analysis of periodontal disease–cancer research to track its development and identify current trends and focal points over the past ten years. This objective is to provide researchers with a comprehensive overview of this research landscape, potentially leading to more in-depth future investigations.

## 2. Materials and Methods

### 2.1. Data Sources and Search Strategy

The Thomson Reuters Web of Science (WoS) Core Collection database was applied in this study for analyzing published research on periodontal disease and cancer. This database extensively covers the medical literature and possesses detailed citation analysis features [25]. Both the Science Citation Index Expanded (SCIE) and the Social Sciences Citation Index (SSCI) editions were included.

**Figure 1:**
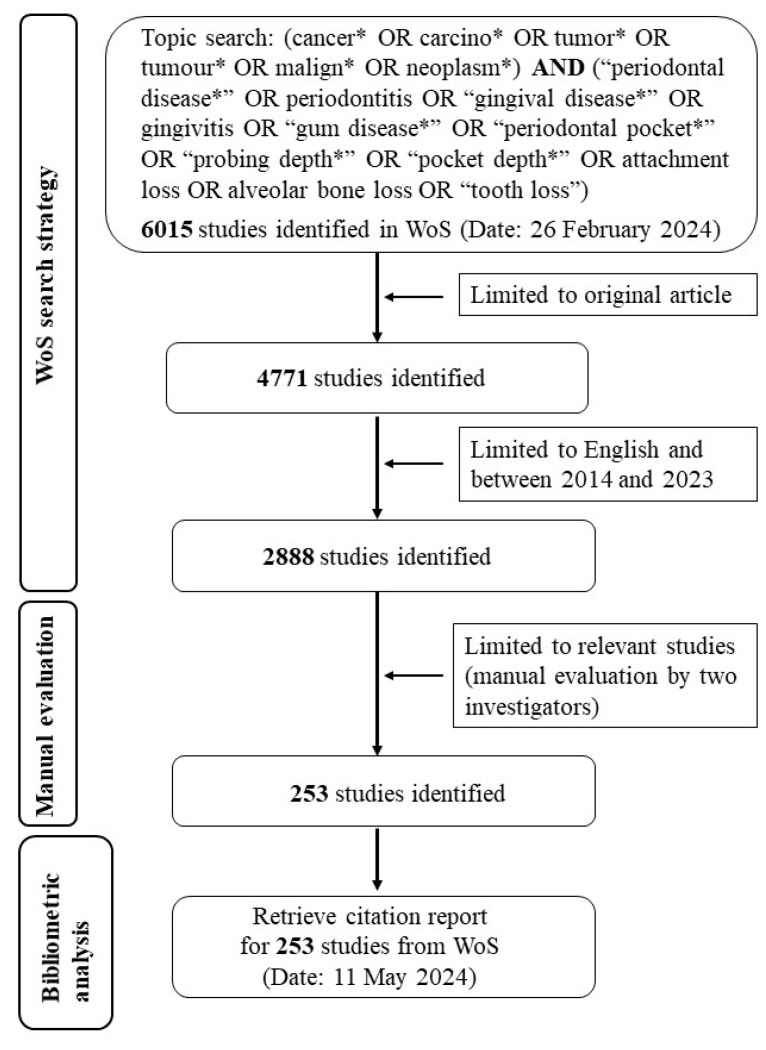
Flowchart illustrating the search strategy and article selection process. The asterisk (*) represents a wildcard for zero or more characters. The initial search yielded 6015 articles, with 253 remaining after the final screening.

The advanced search on WoS included specific terms related to cancer and periodontal disease and their synonyms. The search strategy applied is shown in Figure 1. Searching by “Topic” in the WoS database retrieves all documents with the specified terms in the title, abstract, or author keywords sections.

### 2.2. Data Collection and Processing

This study only considered original articles which were written in English and published between 2014 and 2023. To further assess the relevance of these studies to periodontal disease and cancer, two investigators (YL and SH) manually evaluated these articles by reading the abstracts and full texts. In case of discrepancies, contested articles were retained in this study.

### 2.3. Bibliometric Analysis and Visualization

Bibliometric data regarding authors, affiliations, publication dates, journals, countries, citation counts, keywords, and the H-index of the identified publications were extracted from WoS for further exploration. The H-index, an indicator quantifying an individual’s scientific output [26], was used to assess the academic impact of authors, affiliated institutions, and countries. The Impact Factor from the Journal Citation Reports served as an indicator for assessing journals. The following bibliometric information was generated: publications and their citations over time, the most prolific authors and their affiliations, the leading affiliations and their countries/regions, the most productive journals, the average cites per article (number of citations/number of publications), and the top-cited articles in research on cancer and periodontal disease.

Version 1.6.19 of VOSviewer (Centre for Science and Technology Studies, Leiden University, Leiden, The Netherlands), a free computer program, was applied to generate and visualize the co-authorship network among various countries/regions, and the co-occurrence network of keywords from relevant publications.

## 3. Results

### 3.1. Publications and Their Categories and Study Design

After a rigorous selection process, a total of 253 articles that met the search criteria were included in this bibliometric analysis (Figure 1). This final set of articles represented the most relevant studies available on the topic of periodontal disease and cancer. The most frequent WoS categories for these articles were Oncology (93 articles, 36.76%), Dentistry, Oral Surgery & Medicine (47 articles, 18.58%), Microbiology (32 articles, 12.65%), Public, Environmental & Occupational Health (26 articles, 10.28%), and Multidisciplinary Sciences (22 articles, 8.70%).

Manual classification of these 253 articles by the investigators revealed the following distribution: 118 articles were epidemiological studies examining the associations between periodontal disease as a risk factor and cancer. Sixty-seven articles focused on the relationship between oral microbiota or specific periodontal pathogens or genes and cancers in human subjects. The remaining 68 articles were predominantly in vitro studies, animal studies, or bioinformatic analyses that assessed the biological mechanisms linking periodontal disease/pathogens to tumorigenesis.

### 3.2. Publications and Their Citations

**Figure 2:**
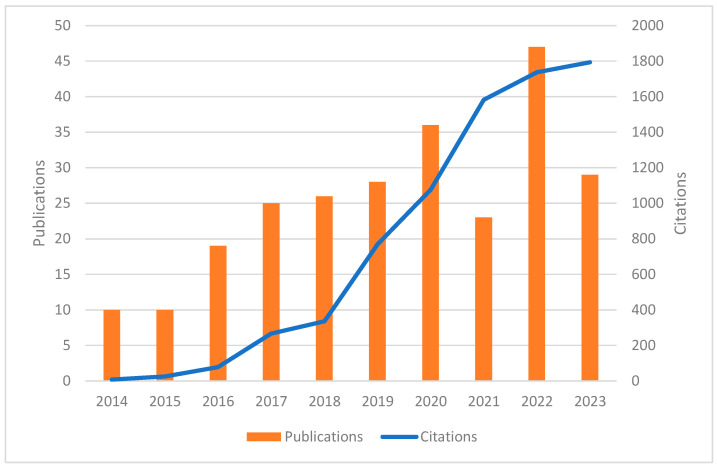
Time trend showing the increasing number of publications and their citations, indicating an upward tendency.

The number of publications demonstrated a clear upward trend over the years, with the highest number observed in 2022, reaching 47 articles, marking the peak of research activity during the analyzed period (Figure 2). Similarly, the corresponding citations of these articles received have also increased over time, with 1794 citations peaking in 2023, demonstrating the increasing recognition and academic engagement with the published studies.

### 3.3. Publications and Citations by Authors, Affiliations, and Countries/Regions

Table 1 lists the most productive authors in the field, along with their respective affiliations, the number of publications, total citations, average citations per article, and H-index. Among the list, Michaud DS ranked first with 12 articles, while Abnet CC had the highest number of citations and average cites, 749 and 74.9, respectively. The ranking of the H-index indicated that Michaud DS, Abnet CC, and Park HR tied for first place with a score of 8.

In terms of affiliations, both the Karolinska Institutet and the National Institutes of Health had the highest number of publications (Table 2). The National Institutes of Health and Harvard University ranked first and second in the number of citations, respectively. Additionally, the Karolinska Institutet had the highest H-index among the institutions. To calculate the H-index of affiliations, we collected all publications that had been produced by researchers affiliated with the institution and gathered the citation count for each publication. The H-index was determined by the highest number (h) such that the institution had at least h papers with h or more citations each.

**Figure 3:**
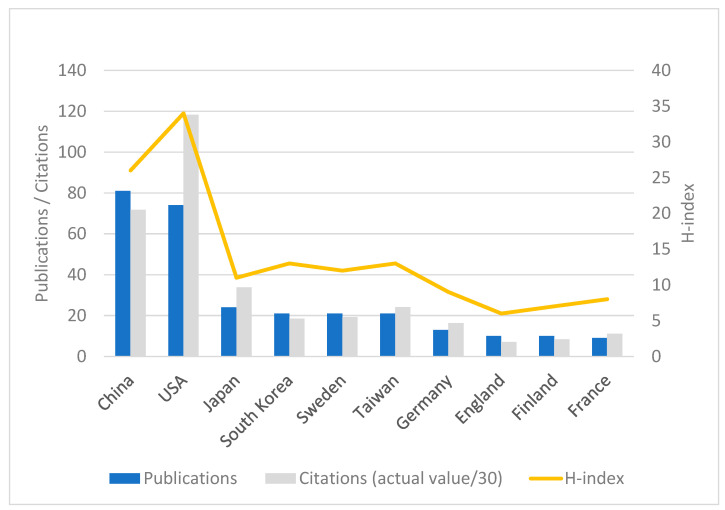
Contributions of different countries/regions to research on the association of periodontal disease and cancer.

The contribution of a country/region to the field of periodontal disease and cancer was assessed using three indicators: the total number of publications, citations, and the H-index (Figure 3). China emerged as the leading country with 81 publications (32.02% of the total). The United States followed with 74 publications (29.25%). Japan produced 24 publications (9.49%), while South Korea, Sweden, and Taiwan each had 21 publications (8.30%). In terms of citation impact, the United States exhibited the highest cumulative citation count of 3549, highlighting the influential nature of its research contributions in the field. China ranked second with 2152 citations. Furthermore, the United States secured the first position with an H-index of 34. China ranked second with an H-index of 26, while South Korea and Taiwan both ranked third with an H-index of 13.

**Figure 4:**
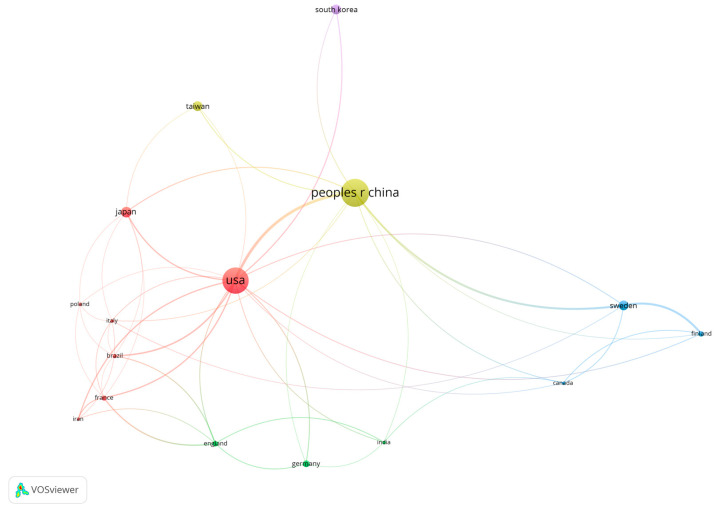
Co-authorship network of different countries/regions, showing international research collaborations.

The co-authorship network of different countries and regions involved in research on periodontal disease and cancer is illustrated in Figure 4. This visualized network used nodes and connecting lines to represent countries/regions and their collaborative relationships, respectively. Larger nodes indicate countries/regions with higher numbers of publications, while thicker connecting lines represent stronger collaborative links between countries/regions. The United States had strong ties with several countries, including China, Japan, and European nations, demonstrating its central role in fostering international research collaborations. The network also highlighted the global nature of research. This widespread collaboration reflected the universal importance of the research topic and the shared interest in advancing scientific understanding in this area.

### 3.4. Publications and Citations by Journals

The most productive journals in the field of periodontal disease and cancer are listed in Table 3, detailing the number of publications, total citations, average citations per article, impact factor (IF), publisher, and category. The International Journal of Cancer published the highest number of articles (12 publications) and gained a strong impact factor (IF = 5.7), indicating its significance in the field. Two journals, Frontiers in Cellular and Infection Microbiology and Scientific Reports, followed with 11 publications each. For average cites, PLoS ONE, Scientific Reports, and Frontiers in Cellular and Infection Microbiology ranked in the top three.

These leading journals listed in Table 3 are central to the dissemination of research on periodontal disease and cancer. Journals like the International Journal of Cancer and Frontiers in Cellular and Infection Microbiology, and Scientific Reports are pivotal due to their high publication and citation counts, indicating their significant influence and reach within the scientific community. The diverse range of categories, from oncology to microbiology and public health, underscores the interdisciplinary nature of this research field, reflecting the varied approaches and methodologies employed by researchers to address the complex interactions between periodontal disease and cancer.

### 3.5. Top Ten Most Cited Articles

The ten most cited articles in the field of periodontal disease and cancer are listed in Table 4, highlighting the significant role of specific oral microbiota in various cancers. Six studies compared the presence of oral microbiota in cancer patients and normal controls using saliva or oral rinse samples [27,28,29] or swab samples from tumor and non-tumor sites [30,31,32]. Three studies assessed *Fusobacterium nucleatum* in human cancer tissues [33,34,35]. Other studies investigated the effects of *Porphyromonas gingivalis* [36] and *F. nucleatum* [34,36] on tumorigenesis using mouse models. The cancers studied included oral cancer [28,29,30,31,32,36], pancreatic cancer [27,35], esophageal cancer [33], and breast cancer [34]. All of these studies applied molecular techniques, such as 16S ribosomal RNA (16S rRNA) gene sequencing and quantitative PCR (qPCR), to characterize oral microbiota or specific bacteria.

The top-cited article [27] demonstrated that specific oral pathogens were associated with an increased risk of pancreatic cancer using a nested case-control design, suggesting a potential role for the oral microbiota in the etiology of pancreatic cancer. The study found that the presence of specific oral pathogens, such as *P. gingivalis* and *Aggregatibacter actinomycetemcomitans*, in pre-diagnosis oral wash samples was associated with an increased risk of pancreatic cancer, while phylum *Fusobacteria* was associated with a decreased risk. The second most cited article by Yamamura et al. [33] quantified *F. nucleatum* DNA in esophageal cancer tissue and adjacent normal esophageal mucosa by qPCR. The presence of *F. nucleatum* DNA was significantly associated with advanced tumor stages and lower survival rates, proposing its role as a prognostic biomarker and its contribution to aggressive tumor behavior. The third most cited article by Parhi, et al. [34] confirmed that *F. nucleatum* colonized breast cancer tissues and promoted tumor growth and metastasis by suppressing immune responses in a mouse model, suggesting that targeting this bacterium could be beneficial in breast cancer treatment.

Gallimidi et al. [36] showed that chronic infection with *P. gingivalis* and *F. nucleatum* promotes oral squamous cell carcinoma in mice through interactions with oral epithelial cells via Toll-like receptors and the IL-6-STAT3 axis. Mitsuhashi et al. [35] found *Fusobacterium* species in 8.8% of pancreatic cancer tissues, with positive patients showing significantly lower survival rates. Articles ranked 6 to 10 (Table 4) investigated the link between the oral microbiome and oral cancer using 16S rRNA sequencing. These studies reported varying species or microbial shifts, notably an increased abundance of *Fusobacteria* in cancer patients’ saliva or cancer sites, except one study [28], which found lower *Fusobacteria* presence in cancer patients’ saliva.

### 3.6. Keyword Analysis of Research in Periodontal Disease and Cancer

**Figure 5:**
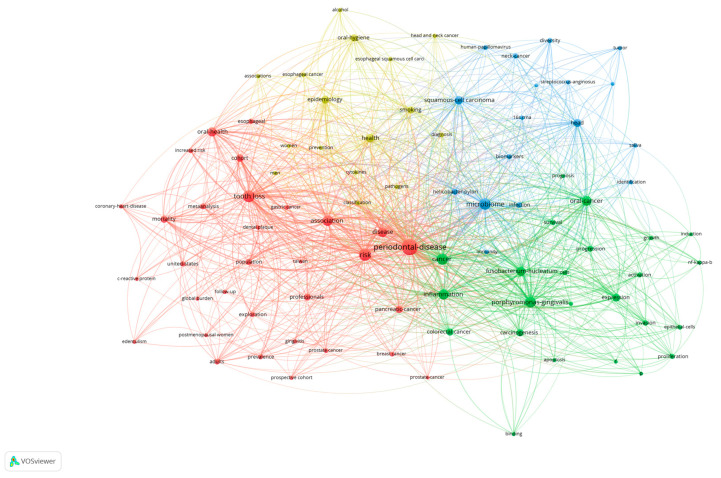
Co-occurrence network of keywords of relevant publication, identifying four distinct clusters.

A bibliometric analysis of this literature by using VOSviewer identified four distinct clusters; each represented a thematic focus area within the research field (Figure 5). The red cluster highlighted studies primarily concerned with periodontal disease and their implications on general health. The keywords in this cluster included periodontal disease, tooth loss, risk, oral health, disease, association, and cohort. Notably, this cluster showed a significant emphasis on how periodontal health impacts systemic conditions, such as coronary heart disease and mortality. Other keywords like meta-analysis, risk, prevalence, and follow-up studies indicated a strong focus on epidemiological methods and longitudinal studies to understand the long-term effects of periodontal disease.

The blue cluster encompassed studies analyzing the microbiome and its association with various carcinomas. The primary keywords include microbiome, squamous-cell carcinoma, neck cancer, head, 16s rRNA, biomarkers, and diversity. This cluster underscored the importance of microbial diversity and specific microbial profiles in the pathogenesis of head and neck cancers. The presence of keywords such as prognosis, identification, and biomarkers suggested that these studies often aimed to identify microbial signatures that could serve as diagnostic or prognostic markers for cancers.

The green cluster focused on inflammation and the roles of periodontal pathogens in carcinogenesis and cancer progression. Keywords in this cluster included *Fusobacterium nucleatum*, *Porphyromonas gingivalis*, inflammation, apoptosis, expression, activation, survival, and various cancer types like colorectal cancer and oral cancer. The terms apoptosis, activation, and expression highlighted research into molecular mechanisms by which these bacteria influenced cancer cell behavior. Additionally, this cluster reflected a deep investigation into the pathways and processes through which chronic inflammation and bacterial infection contributed to cancer development.

The yellow cluster included studies that applied epidemiologic methods to evaluate the association between periodontal disease and cancer. Keywords such as epidemiology, oral hygiene, health, smoking, and prevention indicated a focus on identifying and understanding risk factors and preventative measures. This cluster also included keywords like esophageal cancer and alcohol, pointing to the investigation of lifestyle factors and their interplay with periodontal health in contributing to cancer risk.

## 4. Discussion

This study provides a comprehensive bibliometric analysis of academic original article publications that assessed the association between periodontal disease and cancer from 2014 to 2023. By systematically filtering the initial large pool of articles (Table 1), the authors ensured that only the most pertinent studies were included, thereby enhancing the validity of the findings and conclusions drawn from the analysis. The consistent rise in both publications and citations underscores the dynamic nature of research in the field of periodontal disease and cancer, indicating the growing interest in this research area. The increasing number of publications suggests that more researchers are entering this field, contributing new findings and expanding the body of knowledge. Meanwhile, the rising citations indicate that the research is gaining attention within the academic community, influencing subsequent studies and advancing understanding in this area, signifying the urgent need to understand the links between these two globally prevalent diseases.

The research from the most productive author, Michaud DS, and colleagues revealed a significant impact of oral health on the risk of various cancers. In the Health Professionals Follow-Up Study, severe periodontal disease was linked to a higher risk of non-Hodgkin lymphoma and chronic lymphocytic leukemia/small lymphocytic lymphoma [37]. Tooth loss, often a consequence of severe periodontal disease, was another focal point of Michaud’s research. Studies showed that tooth loss was associated with cancer risks and mortality. For example, fewer teeth were associated with higher colorectal cancer risk in the Nurses’ Health Study [38] and increased pancreatic cancer risk in the Black Women’s Health Study [39]. Moreover, a study using the Health Professionals Follow-up Study data linked tooth loss to higher total cancer mortality, particularly among men with advanced periodontitis [40]. Michaud’s research also explored the role of the oral microbiome in cancer development. The oral microbiome’s composition, particularly the presence of specific pathogenic bacteria, has been investigated in relation to pancreatic cancer among African Americans [41]. The work on lung cancer within the Atherosclerosis Risk in Communities study demonstrated that antibodies to periodontal bacteria, especially those classified as orange complex bacteria, were positively associated with lung cancer risk [42]. Similarly, in the nested case-control study from the CLUE I cohort, antibody level to *A. actinomycetemcomitans* was related to colon cancer risk [43]. These studies suggest that poor oral health and the associated microbial populations may contribute to the racial disparities observed in pancreatic cancer incidence [39,41]. The findings from Michaud DS and colleagues emphasize the need for comprehensive oral health care as a potentially impactful strategy for cancer prevention. Their studies also suggest that oral health can serve as a marker for underlying systemic inflammation and immune dysregulation, further linking it to cancer etiology.

The studies led by Abnet CC spanned multiple cohorts and geographic regions, consistently demonstrating that poor oral health was a significant risk factor for cancers, particularly those of the upper gastrointestinal tract, liver, and pancreas. In both the Finnish cohort study [44] and the Linxian Nutrition Intervention Trial [45,46], greater tooth loss was associated with increased mortality from upper gastrointestinal cancers and increased risks of liver cancer, respectively. Similarly, in the Iran Golestan Cohort Study, significant tooth loss predicted a higher risk of esophageal cancer [47,48] and gastric cancer [48] and increased cancer mortality [49]. Another significant aspect of their research is the exploration of the oral microbiome’s role in cancer risk. Studies conducted in Iran [50], China [51], and the United States [27] highlighted distinct microbial profiles in patients with esophageal squamous cell carcinoma, gastric cardia adenocarcinoma [51], and pancreatic cancer [27,50]. For instance, the presence of specific pathogens like *P. gingivalis* was associated with higher pancreatic cancer risk, suggesting that periodontal pathogens may play a crucial role in carcinogenesis [27]. These studies conducted by Abnet CC and colleagues collectively present a compelling body of evidence linking oral health to the risk of various systemic cancers, emphasizing the broader implications of periodontal disease beyond the oral cavity.

These leading authors in the field of periodontal disease and cancer research exhibit their productivity and impact through publication and citation metrics. Authors like Michaud DS and Abnet CC stand out for their prolific output and high citation counts, indicating their influential roles in advancing the field. The affiliations of these top authors, including prestigious institutions like the National Cancer Institute and Karolinska Institutet, further emphasize the importance of their research environments in fostering significant scientific contributions.

The analysis of the ten most cited articles highlights the significant role of specific oral microbiota in the development, progression, and prognosis of various cancers (Table 4). These studies collectively elucidate how periodontal pathogens and oral microbiota contribute to carcinogenesis. The presence of certain oral pathogens, such as *F. nucleatum* and *P. gingivalis*, was frequently linked to various types of cancers, including pancreatic, esophageal, and oral cancer. These pathogens were also associated with poorer prognosis in cancer patients. Variations in the abundance and diversity of microbial communities have been observed in oral rinse from cancer patients compared to normal controls [27,29] and in cancer tissues compared to non-cancerous sites [30,31,32,33,34,35]. These microbial shifts correlated with cancer patients’ survival rates [33,35], indicating a potential relationship between microbial dysbiosis and tumor progression. Specifically, alterations in the composition of the oral microbiota at different stages of oral squamous cell carcinoma [29] and changes in the abundance of *F. nucleatum* at different stages of esophageal cancer [33] suggested that bacterial dysbiosis might influence tumor development. These studies identified possible mechanisms through which these bacteria may contribute to cancer progression, including interactions with tumor cell surface molecules and suppression of immune responses. These mechanisms suggested that specific periodontal pathogens could serve as biomarkers for assessing cancer risk. Additionally, targeting these bacteria or their interactions with host cells could be a viable therapeutic strategy to inhibit tumor growth and metastasis. The presence of these bacteria in cancer tissues also holds potential as biomarkers for cancer prognosis, aiding in the identification of high-risk individuals.

Notably, the top-cited article by Fan et al. [27] was unique in analyzing oral wash samples collected before cancer diagnosis. This prospective approach provided possible solutions to the questions about whether tumor development or progression alters the microbiota, encouraging further verification through longitudinal studies. Despite the insights provided, several studies within the top ten revealed inconsistencies regarding specific species or microbial shifts associated with oral squamous cell carcinoma [28,29,31,32]. One limitation is their narrow focus on compositional analysis, which may not fully capture the functional dynamics of the microbiome. To address this, some studies have employed functional prediction analysis [29,31,32], revealing an enrichment of pro-inflammatory bacterial attributes indicative of an inflammatory bacteriome. This suggests that integrating compositional and functional analyses of the microbiome could offer a more comprehensive understanding of its role in cancer. These highly cited studies underscore the importance of oral health and oral microbiota in cancer research. They highlight the need for continued exploration into the mechanisms of microbial contributions to cancer, the development of microbiome-based biomarkers, and the potential for microbiota-targeted therapies.

Four key clusters within the research on periodontal disease and cancer were identified by using VOSviewer, each highlighting distinct yet interconnected areas of study (Figure 5). The red cluster focused on the broader implications of periodontal disease on general health, indicating a significant link between oral health and systemic conditions such as coronary heart disease and mortality. The emphasis on keywords like tooth loss, oral health, and mortality suggested that periodontal disease was not only a local oral health issue but also a contributor to systemic health problems. The blue cluster underscored the critical role of the microbiome in carcinogenesis, particularly squamous-cell carcinoma of the head and neck. Keywords such as microbiome, saliva, 16s rRNA, and biomarkers pointed to the importance of microbial diversity and specific bacterial profiles in cancer development. This cluster emphasized the potential for using microbiome analysis as a diagnostic tool and for identifying biomarkers that could aid in early cancer detection and prognosis. In the green cluster, the focus was on inflammation and the involvement of specific bacteria like *F. nucleatum* and *P. gingivalis* in cancer development and progression. Keywords such as inflammation, apoptosis, and oral cancer highlighted the molecular and cellular mechanisms through which these bacteria influence carcinogenesis. This cluster suggested a deep investigation into the pathways by which chronic inflammation and bacterial infection might contribute to cancer, emphasizing the need for targeted therapeutic interventions. The yellow cluster highlighted the epidemiological aspect, exploring the associations between periodontal disease and cancer. The presence of keywords such as epidemiology, oral hygiene, health, and smoking indicated a comprehensive approach to identifying risk factors and preventive measures. This cluster reflected the significance of lifestyle factors, including smoking and alcohol consumption, in the etiology of cancer, reinforcing the importance of public health interventions aimed at reducing these risks.

The insights derived from this bibliometric analysis provide a robust foundation for guiding future research in periodontal disease and cancer. Future studies should adopt an interdisciplinary approach, combining dentistry, microbiology, oncology, and epidemiology to explore the systemic effects of periodontal disease on both oral and general health outcomes. This comprehensive approach will help in understanding the full impact of periodontal health on overall well-being. The findings from the blue cluster highlight the potential of the microbiome as a diagnostic tool. Future research should focus on large-scale microbiome studies to identify specific bacterial profiles associated with different cancer types. Additionally, longitudinal studies are needed to assess how changes in the oral microbiome over time correlate with cancer development and progression. The green cluster emphasizes the role of inflammation and specific bacteria in cancer. The pathogenic role of *F. nucleatum* in cancer progression, as demonstrated in studies [33,34,36,52], further corroborates the findings of our bibliometric analysis that identifies this bacterium as a recurrent focus in high-impact studies. Future research should thoroughly investigate the molecular mechanisms by which *F. nucleatum* and *P. gingivalis* contribute to carcinogenesis. Understanding these pathways could lead to the development of targeted therapies aimed at modulating the inflammatory response or directly targeting these bacteria. The yellow cluster underscores the importance of epidemiological methods in identifying risk factors for cancer. Future studies should focus on large, population-based cohorts to validate the associations between periodontal disease and various cancers. Additionally, public health interventions aimed at improving oral hygiene, reducing smoking, and moderating alcohol consumption should be developed and tested for their effectiveness in reducing cancer risk.

To fully elucidate the causal relationships between periodontal disease and cancer, future research should integrate longitudinal data, as exemplified by the top-cited article [27]. This methodological approach will facilitate the establishment of temporal sequences and the identification of early markers of disease progression, ultimately enhancing prevention and early intervention strategies. Given the diversity in microbial profiles and inflammatory responses, future research should explore personalized medicine approaches. Tailoring preventive and therapeutic strategies based on an individual’s microbiome and inflammatory status could enhance the effectiveness of interventions and improve patient outcomes.

The manual evaluation of articles compensates for the limitations of database filtering, ensuring the inclusion of only the most relevant studies. The bibliometric analysis of this study provides a robust overview of global research trends, identifying key authors, institutions, and countries contributing to the field. By integrating manual evaluations and detailed analysis of highly cited papers, this study addresses some of the existing limitations in bibliometric analyses. However, there are still some limitations to this study. The study relies solely on the WoS database, which might miss relevant studies published in non-indexed journals, although it includes mainstream journal articles. Older articles tend to accumulate more citations over time, potentially skewing the representation of more recent influential research. Despite efforts, some inherent limitations of bibliometric analysis, such as citation bias and the dynamic nature of research trends, remain challenging to fully overcome.

## 5. Conclusions

This bibliometric analysis highlights the growing interest in the link between periodontal disease and cancer, with an increase in research publications and citations over the past decade. Highly cited articles emphasize the role of specific oral microbiota, particularly *F. nucleatum* and *P. gingivalis*, in various cancers, suggesting their potential as diagnostic markers and therapeutic targets. Future research should adopt interdisciplinary approaches to understand the systemic effects of periodontal disease. Large-scale microbiome studies and longitudinal research are needed to identify cancer-associated bacterial profiles and assess temporal changes in the oral microbiome. Investigating molecular mechanisms of bacterial carcinogenesis may lead to targeted therapies. Epidemiological studies on large population-based cohorts are essential for validating associations and developing public health interventions. This study provides a comprehensive overview of global research trends, offering a foundation for future research aimed at improving oral and general health outcomes.

## Figures and Tables

**Table 1:** The most productive authors and their affiliations.

Authors	Publications	Citations	Average Cites	H-Index	Affiliation
Michaud DS	12	348	29.0	8	Tufts Univ, USA
Abnet CC	10	749	74.9	8	National Cancer Institute, NIH, USA
Ye WM	9	316	35.1	6	Karolinska Institutet, Sweden
Park HR	8	298	37.3	8	Pusan National Univ, South Korea
Vogtmann E	8	167	20.9	7	National Cancer Institute, NIH, USA

**Table 2:** The most productive affiliations and their countries.

Affiliations	Publications	Citations	Average Cites	H-Index
Karolinska Institutet, Sweden	19	536	28.2	11
National Institutes of Health, USA	19	901	47.4	10
Harvard University, USA	16	890	55.6	10
Tufts University, USA	13	361	27.8	8
University of Michigan, USA	13	398	30.6	9

**Table 3:** The most productive journals.

Journal	Publications	Citations	Average Cites	IF	Publisher	Category
International Journal of Cancer	12	414	34.5	5.7	Wiley	Oncology
Frontiers in Cellular and Infection Microbiology	11	526	47.8	4.6	Frontiers Media SA	Immunology; Microbiology
Scientific Reports	11	530	48.2	3.8	Nature Portfolio	Multidisciplinary Sciences
BMC Oral Health	9	81	9.0	2.6	BMC	Dentistry, Oral Surgery & Medicine
Cancer Causes Control	9	192	21.3	2.2	Springer	Oncology; Public, Environmental & Occupational Health
Cancers	9	158	17.6	4.5	MDPI	Oncology
International Journal of Molecular Sciences	9	71	7.9	4.9	MDPI	Biochemistry & Molecular Biology; Chemistry, Multidisciplinary
PLoS ONE	8	427	53.4	2.9	Public Library Science	Multidisciplinary Sciences
Cancer Epidemiology Biomarkers Prevention	7	194	27.7	3.7	American Association for Cancer Research	Oncology; Public, Environmental & Occupational Health
Journal of Dental Research	6	174	29.0	5.7	Sage Publications Inc.	Dentistry, Oral Surgery & Medicine

**Table 4:** Top 10 most cited articles.

Rank	Article Title	Citations	Corresponding Authors	Year of Publication	Country/ Region	Journal (IF)	Cancer Type	Bacteria	Reference
1	Human oral microbiome and prospective risk for pancreatic cancer: a population-based nested case-control study	461	Ahn J	2018	USA	Gut (IF = 23)	Pancreatic cancer	Oral microbiome (oral wash)	[27]
2	Human microbiome *Fusobacterium nucleatum* in esophageal cancer tissue is associated with prognosis	271	Baba H	2016	Japan	Clinical Cancer Research (IF = 10)	Esophageal cancer	*F. nucleatum* (cancer tissue)	[33]
3	Breast cancer colonization by *Fusobacterium nucleatum* accelerates tumor growth and metastatic progression	260	Bachrach G	2020	Israel	Nature Communications (IF = 14.7)	Breast cancer	*F. nucleatum* (cancer tissue, animal study)	[34]
4a	Periodontal pathogens *Porphyromonas gingivalis* and *Fusobacterium nucleatum* promote tumor progression in an oral-specific chemical carcinogenesis model	246	Elkin M	2015	Israel	Oncotarget (IF = 5.168)	Oral cancer	*P.gingivalis*, *F. nucleatum* (animal study)	[36]
4b	Association of *Fusobacterium* species in pancreatic cancer tissues with molecular features and prognosis	246	Nosho K	2015	Japan	Oncotarget (IF = 5.168)	Pancreatic cancer	*Fusobacterium* (cancer tissue)	[35]
6	Changes in abundance of oral microbiota associated with oral cancer	240	Albertson DG	2014	USA	PLoS ONE (IF = 2.9)	Oral cancer	Oral microbiome (swabs)	[30]
7	Variations in oral microbiota associated with oral cancer	211	Hu B, Liang JP, Zhang CP	2017	China	Scientific Reports (IF = 3.8)	Oral cancer	Oral microbiome (swabs)	[31]
8	16s rRNA amplicon sequencing identifies microbiota associated with oral cancer, human papillomavirus infection and surgical treatment	202	Guerrero-Preston R, Sidransky D	2016	USA	Oncotarget (IF = 5.168)	Oral cancer	Oral microbiome (saliva)	[28]
9	Oral microbiota community dynamics associated with oral squamous cell carcinoma staging	190	Chang KP, Chang YL	2018	Taiwan	Frontiers in Microbiology (IF = 4)	Oral cancer	Oral microbiome (oral rinse)	[29]
10	The oral microbiota may have influence on oral cancer	185	Zhang CP, Zheng HJ	2020	China	Frontiers in Cellular and Infection Microbiology (IF = 4.6)	Oral cancer	Oral microbiome (swabs)	[32]

## Data Availability

Data are available upon reasonable request.
